# Modified variational autoencoder for inversely predicting plasmonic nanofeatures for generating structural color

**DOI:** 10.1038/s41598-023-30069-1

**Published:** 2023-03-02

**Authors:** Prajith Pillai, Beena Rai, Parama Pal

**Affiliations:** TCS Research, Tata Consultancy Services, Bangalore, 560066 India

**Keywords:** Optics and photonics, Optical materials and structures, Materials for optics

## Abstract

We apply a modified variational autoencoder (VAE) regressor for inversely retrieving the topological parameters of the building blocks of plasmonic composites for generating structural colors as per requirement. We demonstrate results of a comparison study between inverse models based on generative VAEs as well as conventional tandem networks that have been favored traditionally. We describe our strategy for improving the performance of our model by filtering the simulated dataset prior to training. The VAE- based inverse model links the electromagnetic response expressed as the structural color to the geometrical dimensions from the latent space using a multilayer perceptron regressor and shows better accuracy over a conventional tandem inverse model.

## Introduction

**Figure 1:**
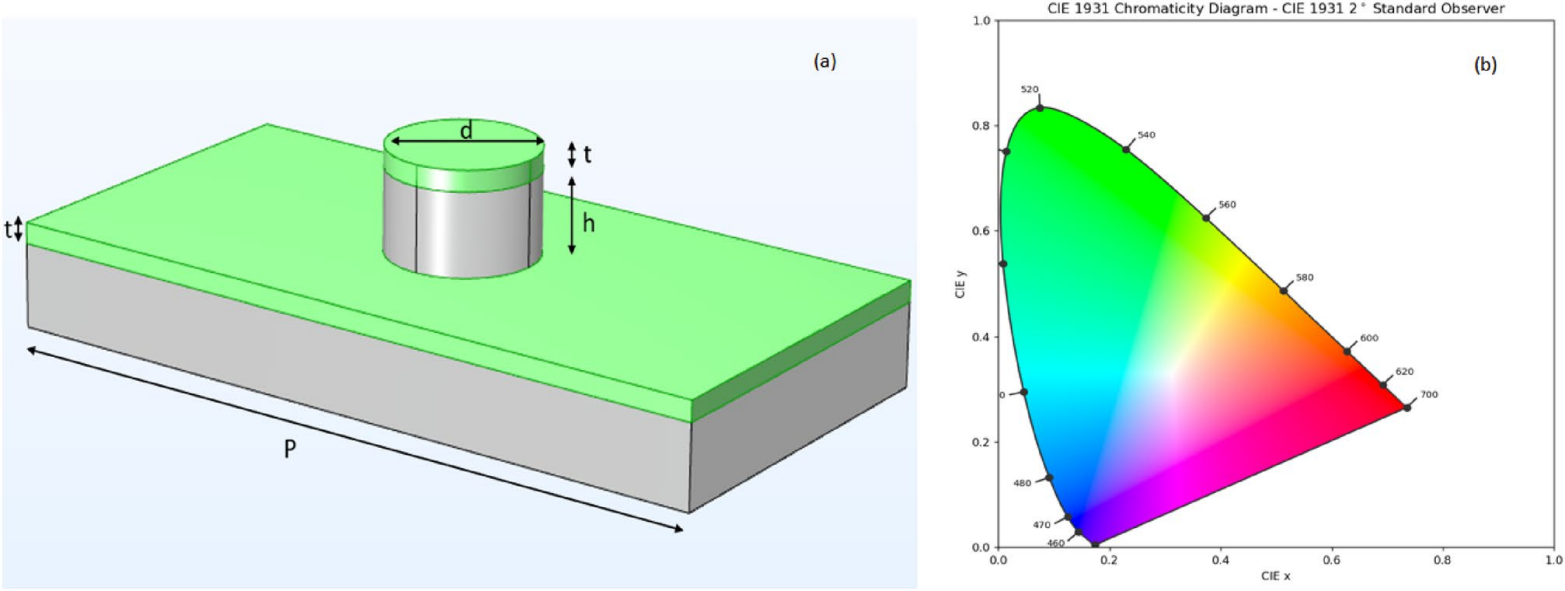
(**a**) Annotated schematic of Al-coated PDMS meta unit (period *P*, pillar diameter *d*, pillar height *h*, and aluminium thickness *t*); (**b**) CIE 1931 chromaticity chart.

**Figure 2:**
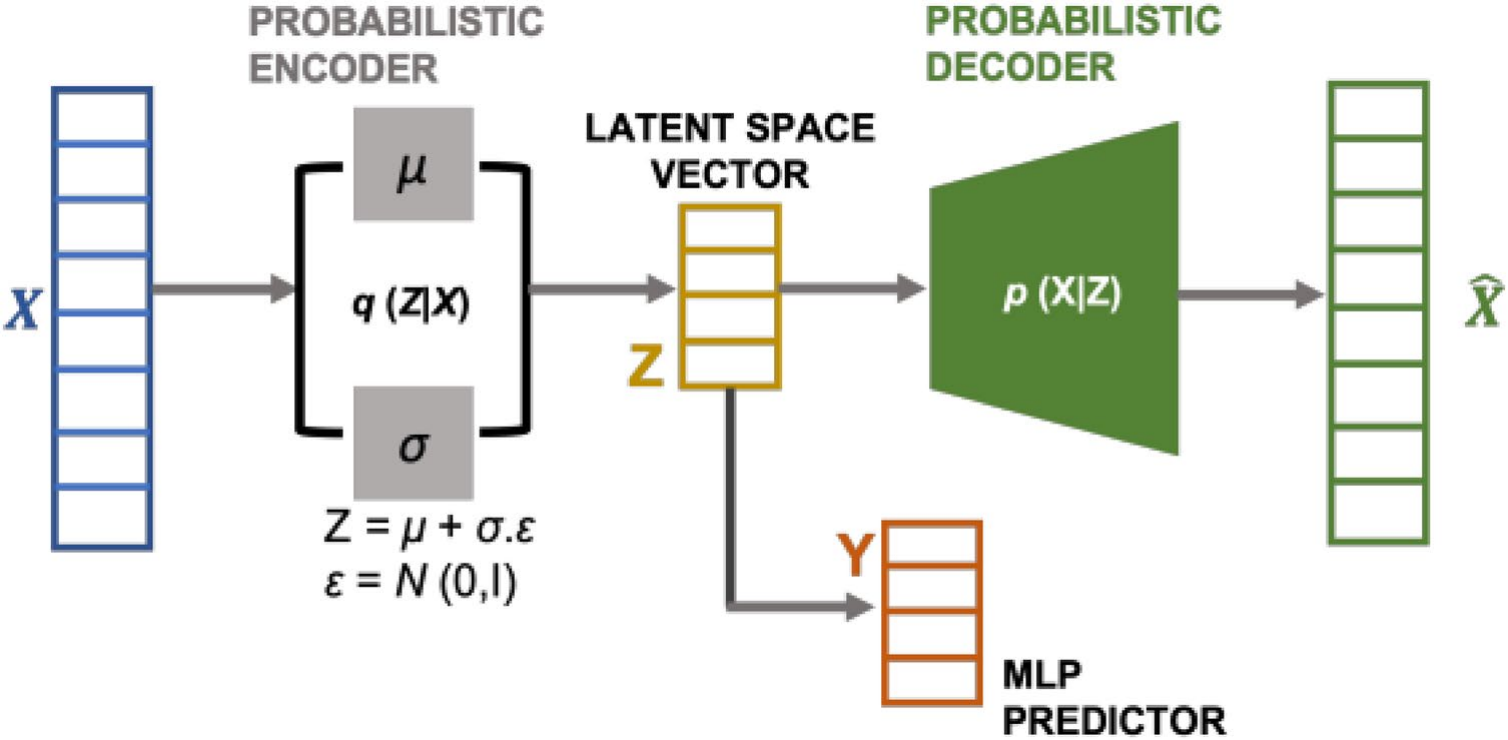
Representative diagram of a modified variational-autoencoder with input X (CIE chromaticity coordinates *x*, *y*) and output Y (*d*, *h*, and *t*).

Plasmonic ‘metasurfaces’ are artificially engineered, two dimensional nanocomposites that have the ability to demonstrate structural color effects^1^. Structural coloration refers to the phenomenon wherein surfaces having topological features smaller than the order of the wavelength of the interrogating light, selectively reflect or transmit specific wavelengths thereby generating color. Owing to their ability to demonstrate selective coloration, plasmonic metasurfaces have been proposed as an ideal material for applications such as intelligent displays for augmented reality/virtual reality devices and high volume consumer electronics. The use of plasmonic effects to achieve structural color as opposed to mixing together different colorants has been shown to be a robust, cost effective scheme to produce colors corresponding to practically any region of the visible spectrum. Metasurfaces in general, comprise of periodic, sub-wavelength unit cells or ‘metaunits’ that interact with incoming radiation in unconventional ways thereby enabling manipulation of physical phenomena (such as reflection, absorption, diffraction) by varying the unit cell dimensions^2^. One example is that of localized surface plasmon resonances (LSPRs) which occur when light is trapped between conductive, nanometer-sized features leading to selective resonant absorption and hybridized reflectance modes resulting in intensely saturated, colored metasurface films. Selective coloration can therefore be achieved by engineering unit cell dimensions appropriately^1–3^. In this work, we describe a model for inversely obtaining meta-unit geometries to achieve structural color produced ‘at will’. We have used the spectral response data (provided in^3^) corresponding to a metasurface made up of a regularly spaced array of polymeric PDMS (polydimethylsiloxane) nanopillars uniformly coated with an aluminum layer (depicted in Figure 1a). We use a filtered, uniformly spaced dataset (which is approximately 10% of the original dataset) for our study. The spectral data can be interpreted via the CIE 1931 color coordinate system wherein any color on the CIE chromaticity chart can be expressed as *x* and *y* coordinate pairs based on the three CIE primaries (Fig. 1b). The human eye has three types of color sensors that respond to different wavelengths and can be visually depicted as a three-dimensional plot. In the CIE 1931 colour space, this 3D plot can be reduced to a 2D plot by using two parameters, namely brightness and chromaticity. More details are available in the supplementary information. Our aim is to inversely retrieve the dimensions of the nanoscale topological features from the observed (perceived) structural color via its corresponding spectral response. This is in contrast to the significantly more tedious process of using a iterative, trial-and-error approach to obtaining the structural feature dimensions for yielding a target color. In recent years, an overwhelming body of work has been dedicated to demonstrating the applicability of data-driven frameworks for designing metamaterials ‘by specification’, i.e., inversely extracting the physical design (of the unit cell typically) that will yield the desired functionality. Deep learning models, owing to their inherent capability for strong generalization, renders them as an ideal choice for mapping morphological and topological features to electromagnetic responses. Early work in this domain has provided only anecdotal evidence for the capability of inverse models to translate to actual fabrication scenarios where the robustness of design models to variations within fabrication tolerances becomes imperative. Of late, nano-photonic inverse design models have been developed using conventional tandem networks with pre-trained decoders as well as convolutional neural network-based autoencoders, the latter having shown to be advantageous for inverse parameter retrieval problems^4,5^. Generative models such as variational autoencoders (VAEs) and GANs, which define the latent space via the mean and standard deviation of a normal distribution and use latent variables to encode the model input(s) to the output(s), have not been sufficiently explored. VAEs and GANs have high diversity compared to tandem networks because of the modal distribution of latent variables and therefore can reconstruct target structures with high accuracies^6^. Conditional VAEs (c-VAEs) have been shown to be advantageous over GANs as they use probability distributions of the initial dataset to generate new data (pertaining to the geometrical/structural details) thereby ensuring that the newly generated data follows the general physics which dictated the original dataset whilst accounting for variances in the parameters defining the geometry of the structure^7^. VAEs are an unsupervised learning method and hence quantifying the latent space (for predicting geometrical dimensions) becomes challenging^8,9^. Some work is also done with modified VAE networks using multi stage training for parameter retrieval^10^. In this work, we describe our inverse model based on a modified VAE regressor (Fig. 2) which provides diversity as well as accuracy in its predictions^11,12^. Our modified VAE has a probabilistic encoder that maps the chromaticity coordinates (*x* and *y*) to the latent space and a probabilistic decoder that maps this latent space back to the *x* and *y* coordinates. We have added a multi-layer perceptron (MLP) from the latent space to the vector space defined by the meta unit’s structural parameters, thereby making the training more versatile by taking into account the reconstruction as well as dimensional prediction(s). The model is thus simultaneously trained with a combined loss function having three components: the mean squared error in the reconstruction of the coordinate space, Kullback-Leibler (KL) divergence (which captures the disparity between the learned distribution and the distribution of the prior network) and the mean squared error in the prediction of dimension through the dense layer added to latent space.

## Results

**Figure 3:**
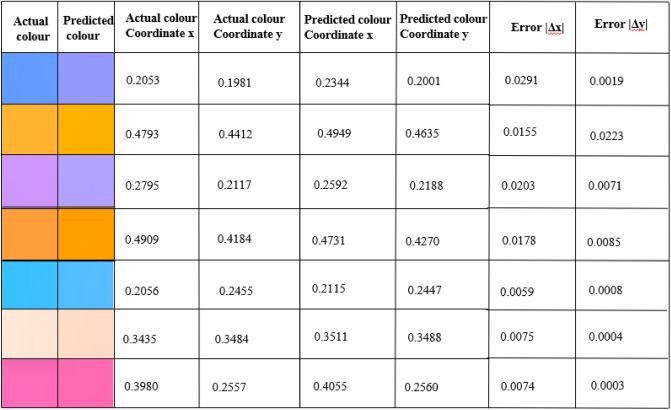
Comparison of randomly selected actual color and model-predicted color and their corresponding CIE 1931 coordinate values.

**Figure 4:**
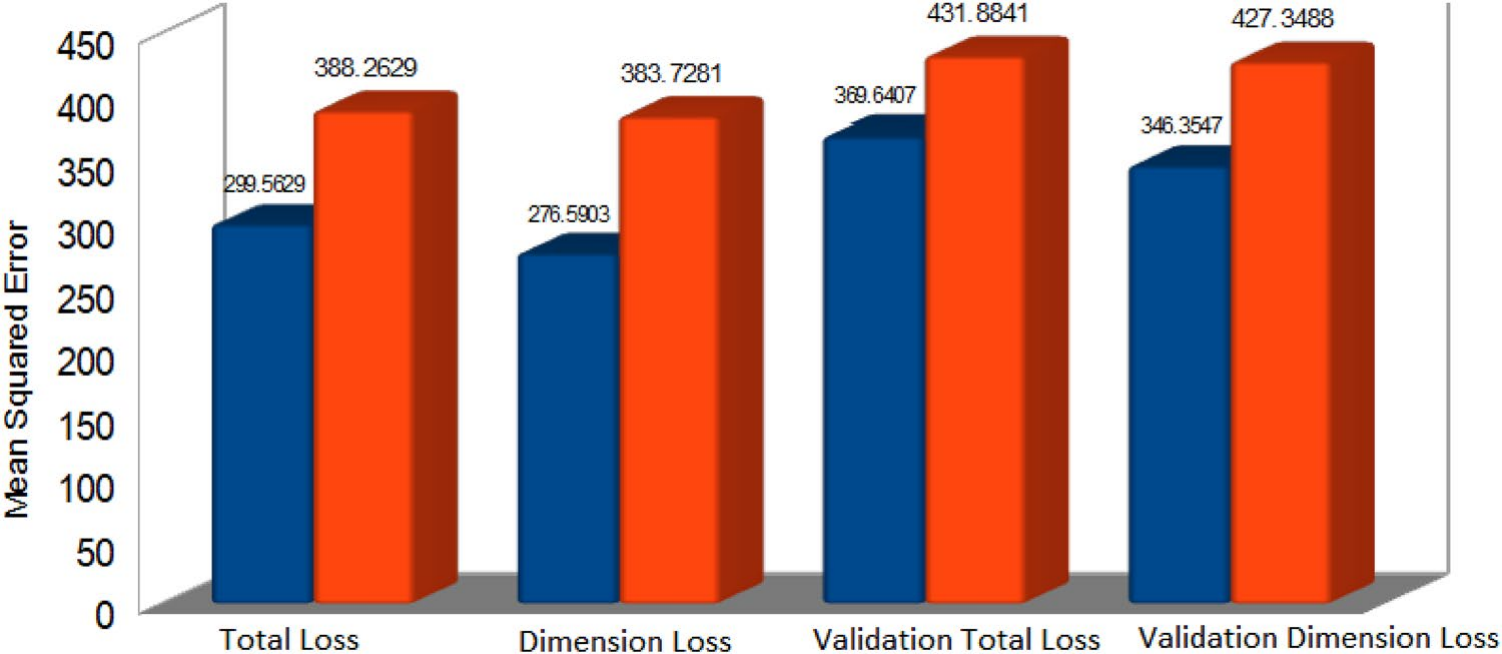
Comparison of training and validation losses for the VAE Regressor (blue) and Tandem Autoencoder (red).

The objective of our modified VAE Regressor is to inversely retrieve geometrical parameters (for a desired structural color expressed via the 1931 CIE color coordinate system) of our plasmonic metasurface comprising of Al-coated PDMS nanopillars. We have added a predictor to the latent space of the VAE and trained the network in a unconventional manner to extract details of the nanopillar for yielding the target color. We train the model using a total loss function that optimizes the model using regeneration accuracy for the decoder and prediction accuracy for the regressor. As the original dataset was spatially distributed in a highly uneven manner, we employed a Euclidean filter to trim (by a factor of 15) the original dataset to reduce clustering. This preprocesing step resulted in a significant improvement of the model performance and the prediction accuracy as compared to when the generative model was trained with the original dataset (details provided in supplementary information). As expected, this step also reduced the computational cost significantly; the training (using Google colab) took approximately 1.5 h with the original dataset which reduced to a little less than 20 min with the reduced dataset in a machine with Intel(R) Core(TM) i7-9750H CPU @ 2.60GHz, 2601 Mhz, 6 Core(s), 12 Logical Processor(s) with 16 GB RAM and 6 GB NVIDIA GeForce RTX 2060 graphics card. The google colab environment had an allocated memory of 12.7 GB RAM and 107.7 GB disk space. The network was further optimized by tuning critical hyperparameters. Our model offers significant improvement over tandem network-based frameworks thereby bringing us closer to a ‘realizable’ approach offering accuracy as well as diversity. This can be readily appreciated from Fig. 3 which depicts color prediction results of randomly selected points from the unseen test data in the vicinity of the achromatic point in the CIE chart. A detailed comparison between the predictions from our model and that of a tandem network is added in the supplementary information file. Our model yields high accuracies for reconstruction (as shown in Fig. 3) as well as for prediction. It outperforms inverse models based on conventional tandem autoencoders in terms of reconstruction accuracy and dimensional prediction (please see supplementary data) and performs well with only 300 training data points. The mean absolute errors while predicting the nanopillar diameter, height and thickness (test set) were 15.97 nm, 14.84 nm, and 4.51 nm respectively.The inversely predicted dimensions done using the trained network is shown in Table 1 for few test cases. A benchmark study of the losses using both tandem network and modified generative VAE regressor is also done for the reduced dataset and the results are shown in Fig. 4. More specifically, the total loss is the combination of the dimensional loss and the reconstruction loss. Total loss and dimension loss corresponds to the training set and the validation total loss and the validation dimension loss corresponds to the validation set. A five-fold cross validation was also done to validate the accuracy of the model on multiple datasets. The t-distributed stochastic neighbor embedding (t-SNE) plots for the input features are added to the supplementary material for better visualization of the features in higher dimension.

**Table 1:** Comparison of actual and predicted dimensions (3 cases from test data).

	Diameter	Height	Thickness
Case1 (Ground Truth)	98	44	92
Case1 (Predicted)	147.35	41.48	92.53
Case2 (Ground Truth)	50	35	50
Case2 (Predicted)	54.21	42.13	50.81
Case3 (Ground Truth)	16	10	20
Case3 (Predicted)	10.72	13.20	19.52

## Discussion

Our demonstration of an inverse model based on a modified VAE regressor shows great promise for retrieving the structural parameters of Al-coated nanopillar meta-units required to generate a desired structural color. A Euclidean filter was applied to the original dataset which was highly clustered. The resultant, evenly spaced set of data points was then used for training the model. The model prediction was better compared to the unfiltered dataset which indicated that for the generative models such as VAEs, smaller, optimally distributed datasets not only significantly improves the prediction accuracy but reduces the overall computational costs as well. A comparative study was also carried out with an inverse model using a non-generative tandem autoencoder using the reduced dataset. The modified VAE regressor model was observed to outperform the traditional tandem networks in terms of the accuracy as well as training time. Thus we can conclude that generative models such as variational auto encoders (VAEs) and generative adversarial networks (GANs) are the ‘go-to’ choice for inverse problems in the area of metamaterials when dataset availability is low, data quality is good and we do not want to compromise on prediction accuracy. We anticipate that the advantages associated with these architectures will play a key role in accelerating the ‘discovery’ of versatile devices based on high-performance functional materials.

## Methods

### Dataset refinement

**Figure 5:**
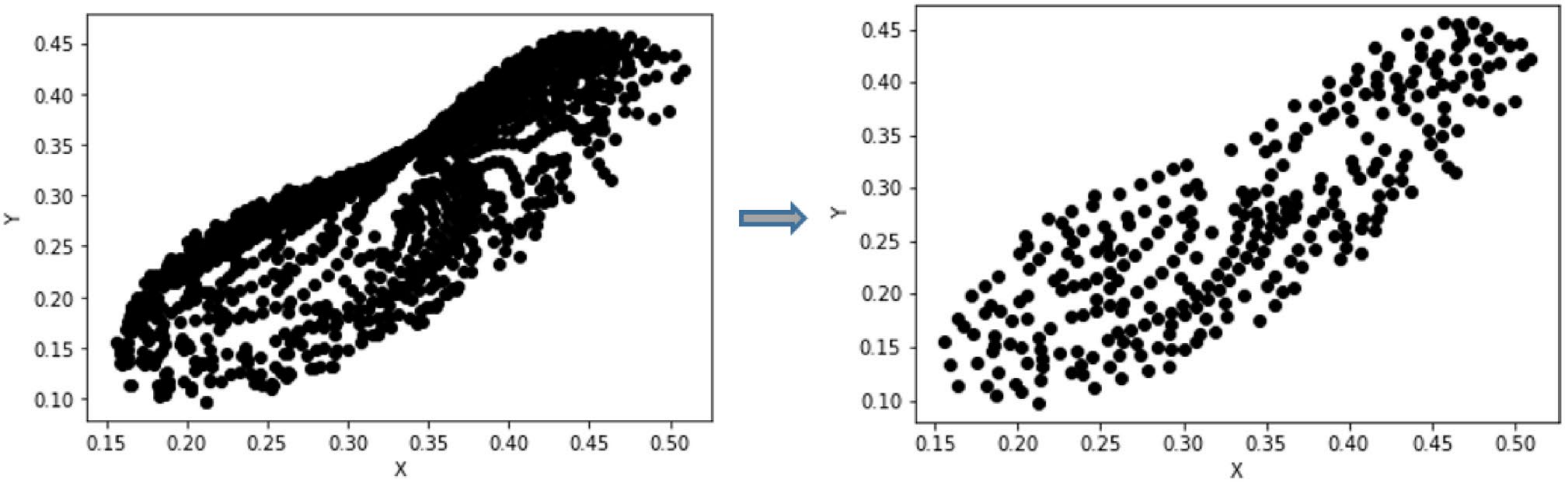
*x* and *y* chromaticity coordinates : original dataset(left), filtered dataset (right).

**Figure 6:**
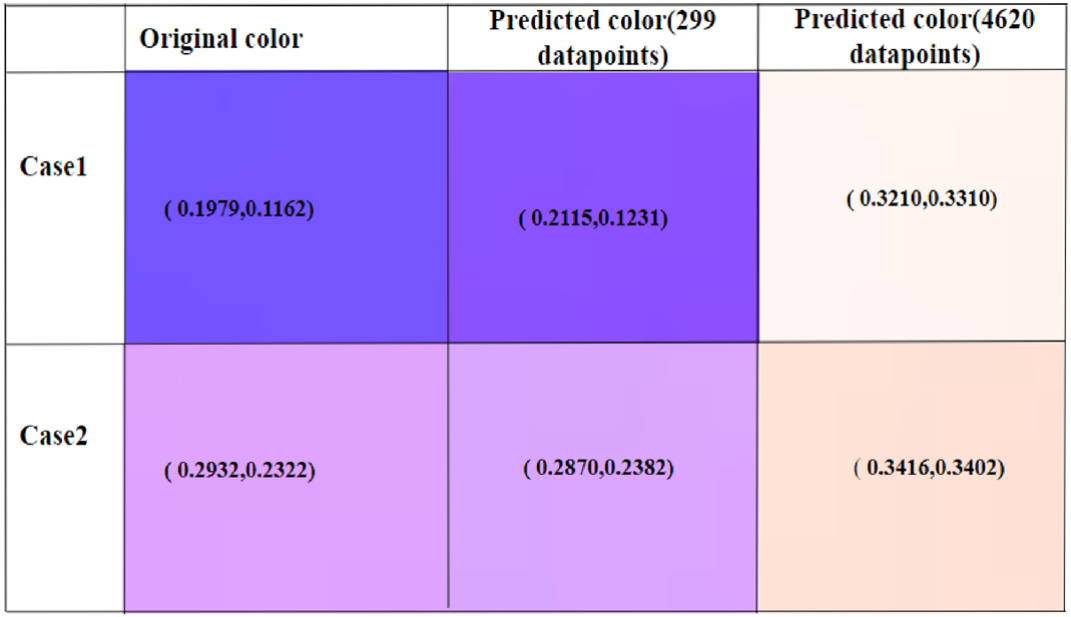
Comparison of colour and coordinates predicted by modified VAE regressor with refined and original dataset.

The nanopillar feature under consideration here generates colors concentrated within an oblong region around the achromatic or ‘white’ point of the CIE chart resulting in uneven clustering and hence insufficient learning owing to the possibility of multiple outputs for similar inputs. To avoid skewing towards this cluster, we evaluated the Euclidean distance between all the points in the coordinate space and rejected all points whose Euclidean distance lay below a threshold set by us. A qualitative study was done wherein 5 threshold values were randomly chosen and the reduction in the clustering was evaluated qualitatively. We chose a threshold value that reduced the dataset to 299 points (shown in Fig. 5). Although this meant a reduction of the dataset size by a factor of nearly 15, it resulted in more evenly spaced points with no clustering and significantly improved the learning for the modified VAE and subsequently, the prediction accuracy, thereby obviating the need to generate large datasets. This can be seen from the comparative study performed by us to illustrate the effect of the dataset reduction and refinement on the generative model’s performance. We understand that in some very special cases involving experimental data corresponding to actual fabricated devices, it may not always be possible to restrict the number of data points and satisfy a uniformly distributed set of data points simultaneously however, the use of an architecture that performs well with limited datasets is of immense advantage in general. Figure 6 shows the colour and coordinate comparison for two cases of unseen test set common to both (original and refined) dataset.The dimension prediction and error in prediction of colour coordinates is added in the supplementary information. The reduced and refined dataset was further applied to both the tandem autoencoder model as well as the modified VAE model for the comparison study of two architectures.

### Model evaluation

Our modified VAE network takes in the CIE *x* and CIE *y* chromaticity coordinates as inputs for the encoder. The output B of the MLP (linked to the latent space) yields the meta-unit (nanopillar) dimensions. We have a distribution *z* for the latent space and we try to generate a sample *x* containing CIE coordinates from this distribution which is represented by *p(x|z)*. From Bayes theorem we have

1 $$\begin{aligned} {p(x|z)} =\frac{{p(z|x)}*{p(x)}}{{p(z)}} \end{aligned}$$

We approximate *p*(*z*|*x*) using a variational posterior represented as *q*(*z*|*x*) and the difference in the distribution is evaluated using the Kullback-Leibler (KL) divergence loss. Therefore, our objective becomes a minimisation problem wherein;

2 $$\begin{aligned} {min KL(q(z|x)||p(z|x))} \end{aligned}$$

The above minimisation problem is equivalent to the following maximisation problem

3 $$\begin{aligned} E_{q(z|x)} log p(x|z) - KL(q(z|x)||p(z)) \end{aligned}$$

where the first term is the reconstruction likelihood for the coordinate space and the second term represents the learned distribution’s similarity to the prior distribution. The combined loss for the variational autoencoder modifies to,

4 $$\begin{aligned} Loss = L(x,{\hat{x}}) + \Sigma _j KL(q(z|x)||p(z)) \end{aligned}$$

**Figure 7:**
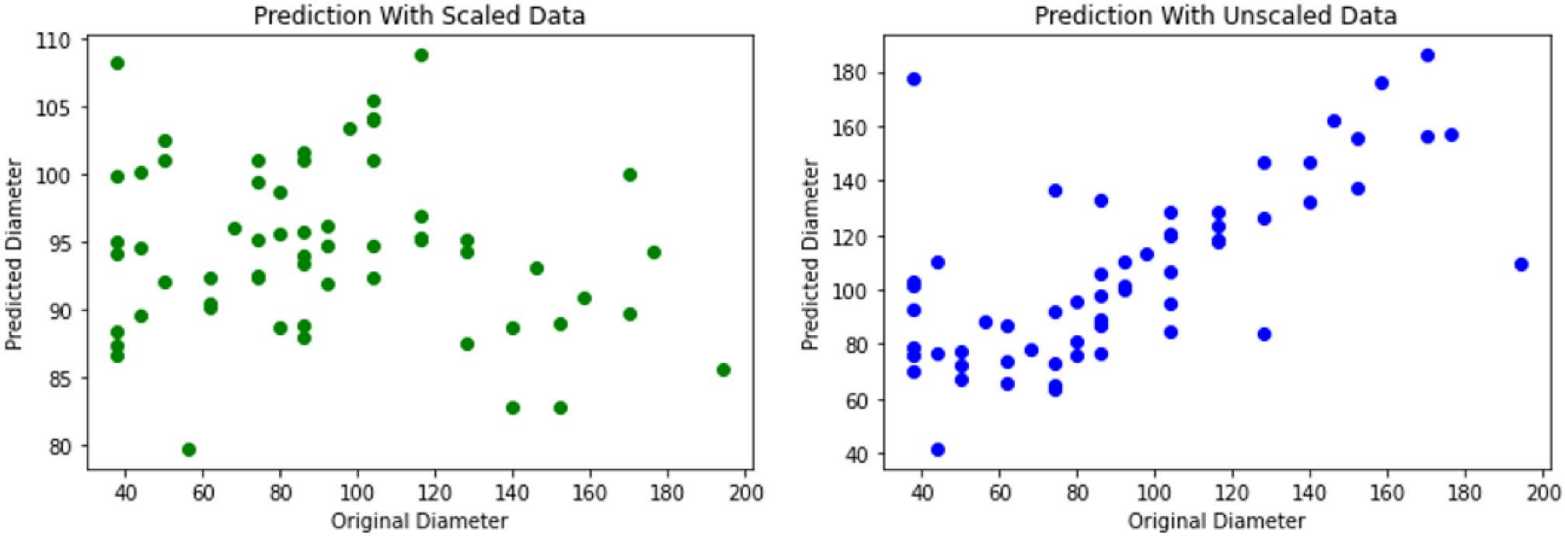
Prediction of pillar diameter ‘*d*’ for scaled (left) and unscaled (right) data.

Now, we add an MLP regressor to the latent space which tries to recreate the dimensions corresponding to the colour coordinate space. This loss gets added to the combined loss giving the total loss equation as shown below

5 $$\begin{aligned} Loss_{total} = L(y,{\hat{y}})+L(x,{\hat{x}}) + \Sigma _j KL(q(z|x)||p(z)) \end{aligned}$$

where the first term is the dimension loss, the second is the reconstruction loss and the third term is the KL divergence loss. The training dataset is a collection of tensors encoding the nanopillar diameter *‘d’*, height *‘h’* and thickness *‘t’* of the Al coating along with the corresponding structural color coordinates. We performed a feature selection exercise to feed the most relevant features into the model thereby avoiding overfitting from using highly correlated predictors. A correlation matrix (shown in Table 2) was computed for the structural parameters (*d*, *h*, and, *t*) which showed minor correlation between the pillar diameter *d* and aluminium thickness *t* but almost zero correlation for the other parameter pairs indicating that all three parameters could be used for our model. We also conducted a study on the effect of scaling on the reduced dataset. The data for the geometrical variables was scaled using the ‘MinMaxScaler’ from Python scikit-learn 1.1.2. The scaled data does not perform well compared to the unscaled data for predicting the meta unit’s geometrical parameters. This can be seen in Fig. 7 where the actual and predicted diameters show a linear relationship with the initial hyperparameters. Hence, we proceeded with unscaled data for our model. The total dataset was split in the ratio 80:10:10. 10 percentage data was used as unseen test set for performance evaluation. The model was trained for 2500 epochs with an early stopping criteria by monitoring the validation loss. The model was run on Google colab.

**Table 2:** Correlation matrix for the geometric dimensions.

	Diameter	Height	Thickness
Diameter	1.0000	$-\,\,$0.0324	$-\,\,$0.1977
Height	$-\,\,$ 0.0324	1.0000	$-\,\,$0.0486
Thickness	$-\,\,$ 0.1977	$-\,\,$0.0486	1.0000

### Hyperparameter tuning

To optimize the number of network layers and neurons (in each layer), the reconstruction loss, dimensional loss, reconstruction validation loss and dimensional validation loss were calculated initially by varying the number of layers from 1 to 5 (for 512 neurons). Subsequently, the neurons in each layer were varied as 256, 512, and 1024. We observed that the lowest validation losses corresponded to 2 dense layers having 512 neurons each. We also tuned model hyper-parameters including the learning rate (varied as 0.1, 0.001, 0.001, 0.0001), activation functions (Sigmoid, Tanh, ReLU, SoftPlus), optimizers (SGD, Adam, RMSprop), latent space dimensions (varied as 3, 5, 10, 100) and regularization (L1, L2). The best performance was obtained with an Adam optimizer (learning rate of 0.01), and ReLU activation function for the encoder, decoder, and predictor. The final model had a latent space dimension equal to 10 and L2 regularization with lambda (regularization parameter) of 0.0001. The loss tables for the hyper-parameter tuning is provided in the supplementary information. Post hyper-parameter tuning, the model reconstruction and dimensional validation losses were 345.1534 and 329.7767 respectively.

## Supplementary Information

Supplementary Information.

## Data Availability

The datasets analysed during the current study are available at: 10.5281/zenodo.5102770.
