# Supplementary material for: Modified variational autoencoder for inversely predicting plasmonic nanofeatures for generating structural color
Source: Sci Rep. 2023 Mar 2;13:3536. doi: 10.1038/s41598-023-30069-1 (PMC9981595; doi:10.1038/s41598-023-30069-1)
Supplement: Supplementary file 1 — Supplementary Information. [file 41598_2023_30069_MOESM1_ESM.pdf]

Supplementary material for Scientific Reports

Chromaticity chart CIE 1931

Human eye has three types of colour sensors that respond to different ranges of wavelength. Full plot is a 3 dimensional figure. However the concept of colour can be divided into two parts brightness and chromaticity. The tristimulus values associated with a color space can be conceptualized as amounts of three primary colors in a tri-chromatic, additive color model. Based on the tristimulus values X,Y,Z we determine the x and y in CIE 1931 colour space

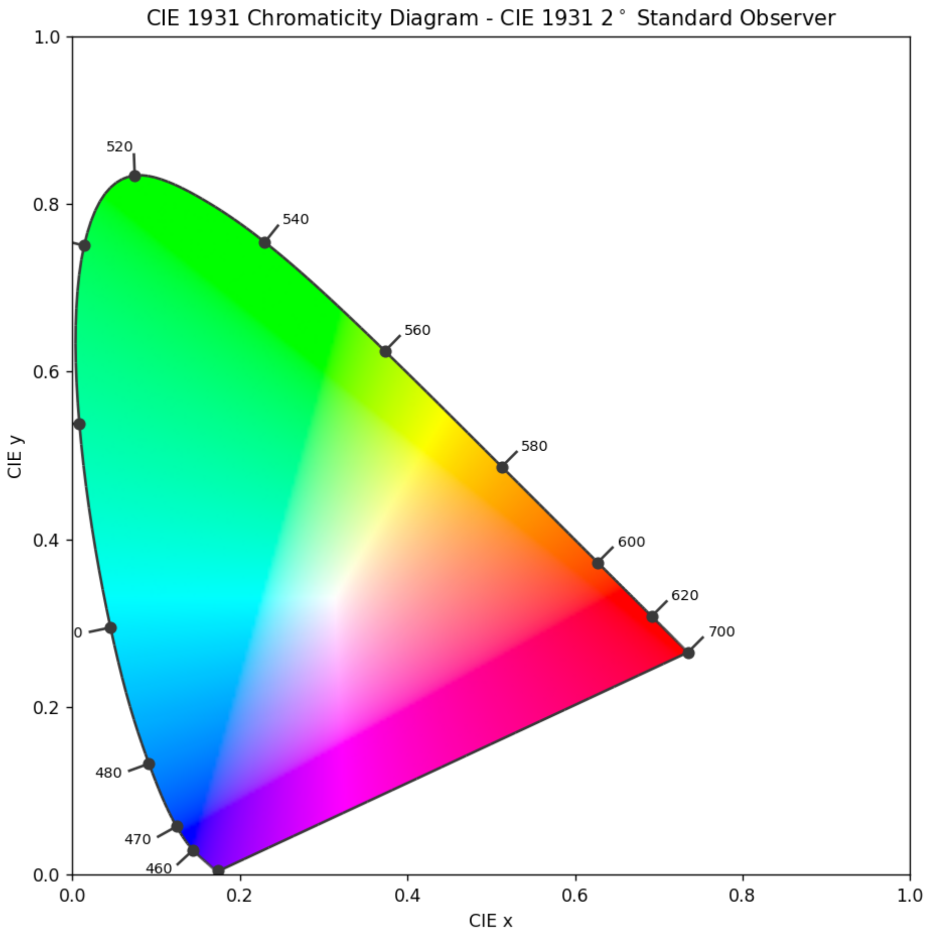

Figure S1. *CIE 1931 chromaticity chart*

Tandem network results

Mean absolute error for the test set using tandem network:
Diameter : 20.17123374938965
Height :15.188144810994466
Thickness : 4.436341190338135

Loss values for tandem network after 2500 epochs:
Loss: 388.2629
Dimensions_loss: 383.7281
Val_loss: 431.8841
Val_dimensions_loss: 427.3488

Table 1. *Predicted x and y coordinate in chromaticity chart using both tandem and modified VAE regressor for unseen test set.*

tandem x	tandem y	original x	original y	VAE x	VAE y
0.311421	0.235173	0.205312	0.198158	0.234471	0.20011
0.317166	0.246658	0.279568	0.211715	0.259235	0.218842
0.346766	0.306073	0.490953	0.41848	0.473144	0.42703
0.312841	0.238013	0.205648	0.245515	0.211565	0.24471
0.38242	0.361473	0.479368	0.441238	0.494953	0.463584
0.38242	0.361473	0.343577	0.348467	0.351133	0.348894
0.29986	0.212065	0.398054	0.255731	0.405513	0.256062

Table 2. *Colour prediction using tandem network for unseen test set*

Actual colour	Predicted Colour Tandem	Actual colour Coordinate x	Actual colour Coordinate y	Predicted colour Coordinate x	Predicted colour Coordinate y
		0.205311901	0.198157787	0.311421	0.235173
		0.47936778	0.441238057	0.317166	0.246658
		0.279568389	0.211715401	0.346766	0.306073
		0.490952676	0.418480077	0.312841	0.238013
		0.20564836	0.245515287	0.382420	0.361473
		0.343576543	0.34846719	0.382420	0.361473
		0.39805409	0.255730626	0.299860	0.212065

Hyperparameter tuning for modified VAE Regressor

Table 3(a). Tuning the number of layers

	1 layer	2 layer	3 layer	4 layer	5 layer
loss	377.4251	299.5629	718.0974	683.9950	706.9542
Dimensional loss	354.3940	276.5903	713.8491	678.8408	703.2244
validation loss	388.1904	369.6407	629.7440	651.8133	647.4295
Dimensional validation loss	366.1104	346.3547	624.1654	647.5333	642.7325

Table 3(b). Tuning the number of neurons in each layer

	256 neurons	512 neurons	1024 neurons	2048 neurons
loss	389.2942	299.5629	335.5479	747.2715
Dimensional loss	369.5289	276.5903	312.4278	741.5340
validation loss	429.9585	369.6407	415.3737	653.6366
Dimensional validation loss	410.8350	346.3547	393.2337	647.4291

Table 3(c). Other hyperparameters tuned

	Variations
Learning rate	0.1, 0.01, 0.001, 0.0001
Activation function	Sigmoid, Tanh, Relu, Softplus
Latent space dimension	3, 5, 10, 100
Optimizer	SGD, Adam, RMSprop
Regularization	L1,L2

Table 4. Dataset size reduction and corresponding losses

Dataset size	299	493	1751	4623 (original)
loss:	396.3033	362.3859	691.4976	872.5547
predictor_loss:	347.9954	348.0761	688.7747	865.8214
val_loss:	405.404	384.6989	699.6620	835.2282
val_predictor_loss:	354.1393	370.3376	697.0013	828.7054

Table 5. Comparison of the predicted and actual dimensions and color coordinates by the modified VAE regressor model trained with refined dataset and original dataset.

	x	y	Diameter(nm)	Height (nm)	Thickness (nm)	|Δx|	|Δy|
Actual case 1	0.197982164	0.116228363	50	75	20		
Predicted case 1 (model trained using 299 datapoints)	0.21158886	0.123193234	64.958626	69.728645	15.4090805	0.01360	0.00696
Predicted case 1 (model trained using 4620 datapoints)	0.32104903	0.33106098	82.881	63.673	20.235	0.12306	0.21483
Actual case 2	0.293216814	0.2322456	80	60	12		
Predicted case 2 (model trained using 299 datapoints)	0.2870228	0.23828924	73.756805	69.18728	12.70443	0.00619	0.00604
Predicted case 2 (model trained using 4620 datapoints)	0.34169918	0.34020728	129.009	67.983	21.524	0.0484	0.1079

Modified Variational Autoencoder:

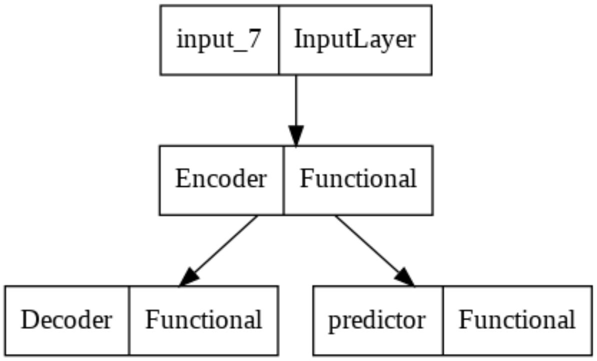

Figure S2. *Architecture of Modified VAE Regressor (python generated)*

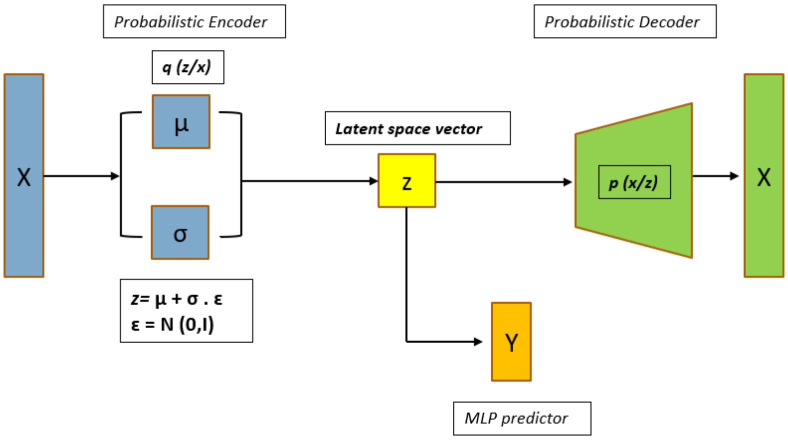

Figure S3. *Modified VAE Regressor (MLP layer connected to latent space)*

Table 6. 5-fold cross validation performed for the final model

fold	total_loss_train	dimension_loss_train	total_loss_val	dimension_loss_val	total_loss_test	dimension_loss_test
1	155.3551	126.0019	98.91866	69.31969	330.3206	298.4203
2	203.603	173.2991	114.9542	86.04119	350.5829	319.6884
3	158.9057	129.6515	101.9395	72.28265	349.5052	319.8111
4	161.5399	131.9396	100.6169	71.58888	436.7476	407.6309
5	91.8923	63.93088	342.3434	314.6055	428.2547	400.6949

<u>t-SNE plots for the input features:</u>

Table 7. *Original dataset format*

X	Y	diameter	height	thickness
0.352166386	0.360159178	38	35	10
0.367039805	0.379699487	38	40	10
0.387610341	0.401142412	38	45	10
0.403248996	0.401599177	38	50	10

a. t-sne plot for coordinates (fig 5):

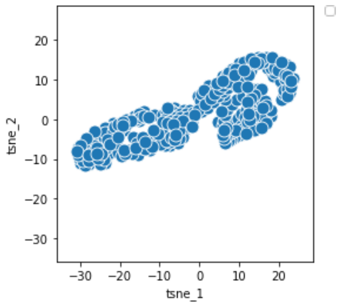

Figure S4. *t- sne plot for figure 5 in main text (x and y coordinates)*

b. t-sne plot for dimensions:

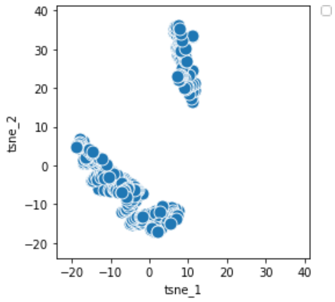

Figure S5. *t- sne plot for dimensions (diameter, height and thickness)*

The t-SNE for the dimension shows 3 groups. The height and thickness being in the same range causes the two groups to be close to each other.

c. t-SNE plot combined (dimensions+ coordinates)

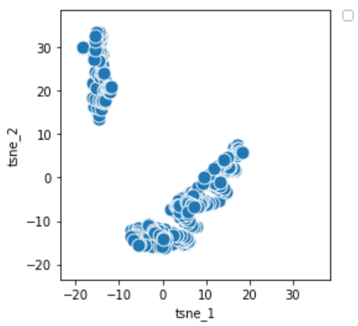

Figure S6. *t- sne plot for dimensions and coordinates together(diameter, height and thickness, x coordinate, y coordinate)*

d. t-SNE plot for fig 7 in the main text

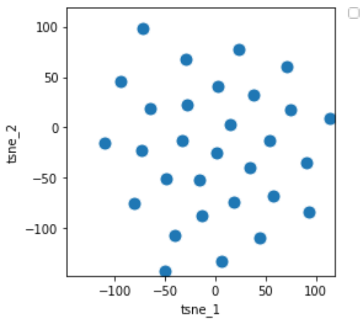

Figure S7. *t- sne plot for figure 7 in main text (predicted and actual diameter)*
